# African cassava whitefly, *Bemisia tabaci*, cassava colonization preferences and control implications

**DOI:** 10.1371/journal.pone.0204862

**Published:** 2018-10-09

**Authors:** Andrew Kalyebi, Sarina Macfadyen, Hazel Parry, Wee Tek Tay, Paul De Barro, John Colvin

**Affiliations:** 1 National Crops Resources Research Institute, Kampala, Uganda; 2 Mikocheni Agricultural Research Institute, Dar es Salaam, Tanzania; 3 CSIRO, Clunies Ross St, Acton, ACT, Australia; 4 CSIRO Ecosciences Precinct, Brisbane QLD, Australia; 5 Natural Resources Institute, University of Greenwich, Chatham Maritime, Kent, United Kingdom; Chinese Academy of Agricultural Sciences Institute of Plant Protection, CHINA

## Abstract

Cassava is a staple food for people across sub-Saharan Africa. Over the last 20 years, there has been an increased frequency of outbreaks and crop damage in this region caused by the cassava-adapted *Bemisia tabaci* putative species. Little is known about when and why *B*. *tabaci* adults move and colonize new cassava crops, especially in farming systems that contain a mixture of cultivar types and plant ages. Here, we assessed experimentally whether the age and variety of cassava affected the density of *B*. *tabaci*. We also tested whether the age and variety of the source cassava field affected the variety preference of *B*. *tabaci* when they colonized new cassava plants. We placed uninfested potted “sentinel” plants of three cassava varieties (Nam 130, Nase 14, and Njule Red) in source fields containing one of two varieties (Nam 130 or Nase 14) and one of three age classes (young, medium, or old). After two weeks, the numbers of nymphs on the sentinel plants were used as a measure of colonization. Molecular identification revealed that the *B*. *tabaci* species was sub-Saharan Africa 1 (SSA1). We found a positive correlation between the density of nymphs on sentinel plants and the density of adults in the source field. The density of nymphs on the sentinels was not significantly related to the age of the source field. *Bemisia tabaci* adults did not preferentially colonize the sentinel plant of the same variety as the source field. There was a significant interactive effect, however, between the source and sentinel variety that may indicate variability in colonization. We conclude that managing cassava source fields to reduce *B*. *tabaci* abundance will be more effective than manipulating nearby varieties. We also suggest that planting a “whitefly sink” variety is unlikely to reduce *B*. *tabaci* SSA1 populations unless fields are managed to reduce *B*. *tabaci* densities using other integrative approaches.

## Introduction

Cassava (*Manihot esculenta*, Euphorbiaciae) is the third most important source of calories in the tropics, after rice and corn [1]. Consumed widely in Africa, Asia, and Latin America, the starchy root crop is grown in 102 countries around the world and provides food security and income to farming communities [2,3]. More than 800 million people use cassava globally and over 200 million of these live in sub-Saharan Africa [1,4]. Although the African continent contains most (66%) of the global cassava growing area, cassava productivity in Africa (8.8 t/ha) is much lower than the world average (10.95 t/ha) [5] and the average in Asia (16.8 t/ha) [6].

One reason for the low productivity of cassava in Africa is that it is attacked by numerous insect pests and diseases. Of the invertebrate pests that attack cassava in East Africa, the whitefly, *Bemisia tabaci* (Hemiptera: Aleyrodidae) is among the most challenging to control. *Bemisia tabaci* is recognized as a pest species complex [7–10], which means that multiple biologically distinct species exist within the species complex but cannot be readily differentiated due to the lack of distinct morphological characters. The *B*. *tabaci* cryptic species complex is agriculturally significant because members within this pest complex are important vectors for a number of plant viruses including begomoviruses, which are the most devastating group in the tropics [11–13]. In Uganda, several *B*. *tabaci* putative species have been recorded on cassava, including Sub-Saharan Africa 1 (SSA1), SSA2, and Indian Ocean [14–16]. Of these species, SSA1 transmits viruses that cause the two most devastating diseases of cassava, cassava mosaic disease (CMD) and cassava brown streak disease (CBSD). The CMD and CBSD greatly reduce yields and compromise the quality of cassava [17–19]. Many CMD-susceptible cassava varieties produce few or no tubers depending on the severity of the disease and the age of the plant at the time of infection. Phloem-feeding by *B*. *tabaci*, indirect damage caused by sooty mold, and transmission of plant viruses can cumulatively reduce yields by up to 80% [20].

Over the last 20 years, there has been an increase in the frequency of outbreaks of indigenous sub-Saharan African members of the *B*. *tabaci* complex in the cassava growing regions of East Africa [21,22], with only a limited understanding of the ecological factors driving population peaks in *B*. *tabaci* [23]. Thus, there is no consensus on managing African cassava *B*. *tabaci*. Instead, the breeding of new cassava cultivars that are resistant or tolerant to CMD and CBSD continues to be the main approach used to limit cassava yield losses. An estimated five improved cultivars are currently in use in Uganda [24,25], plus numerous local ecotypes that vary in yield and general tolerance to pests and diseases. Although the improved cultivars are generally preferred by farmers because of higher yields, early maturity, and greater resistance to diseases [26–28], many smallholder farmers’ fields contain a mixture of varieties either as a mixed planting in one field or planted in adjacent fields. Cassava *B*. *tabaci*, therefore, have several choices when colonizing new plants.

Certain factors may influence *B*. *tabaci* population growth or lead to adult preferences when colonizing a new plant. Although *B*. *tabaci* adults are known to engage in long-distance migration [29–31], not all movement to new plants is in response to reduced availability of feeding and oviposition sites [32]. The *B*. *tabaci* may also preferentially select host plants on which to oviposit via small-scale movements between plants within a field [33,34]. Understanding the preferences of *B*. *tabaci* to oviposit on different cassava varieties and plants of different ages may therefore lead to new management strategies that disrupt the colonization process. These strategies may include alternating the variety planted, changing the timing of planting, or increasing the distance of a new crop from infested fields, all of which could reduce *B*. *tabaci* population growth and ultimately the transmission of diseases.

We designed a manipulative field study to test two research questions. First, does the population density of *B*. *tabaci* differ as a factor of variety or plant age class? We know that in a laboratory setting, older cassava plants are less suitable hosts [35,36]. To test this, we compared the starting density of *B*. *tabaci* adults in fields of different ages (young, medium, or old) that were planted with a single cassava variety (Nam 130 or Nase 14).

Second, does the age and variety of the plant *B*. *tabaci* is reared on (its “source” plant) correlate with its preference when colonizing a new cassava plant? We tested this using the same fields as used for the first research question to act as “source” populations into which we introduced “sentinel” plants (varieties Njule Red, Nam 130, and Nase 14) and investigated the relationship between (i) the density of nymphs on sentinel plants and the density of adult *B*. *tabaci* in the source population, (ii) the density of nymphs on sentinel plants and the age of cassava at the source, and (iii) the density of nymphs on a sentinel variety that matched the source. We predicted that if a cassava source field supported high numbers of adult *B*. *tabaci* then the sentinel plants in that field would have high numbers of nymphs. We also expected medium age fields to be the best source for colonizers, followed by young and finally old fields. We also hypothesized that the density of nymphs on a sentinel variety that matched the source would be higher than when the sentinel did not match the source (i.e. adults preferred to oviposit on a familiar variety).

## Materials and methods

### Source and sentinel plants

Cassava fields of two source varieties (Nam 130 and Nase14) and three different age categories–young, 2–3 months after planting (m.a.p.); medium, 4–6 m.a.p.; and old, 7–9 m.a.p.–were selected from existing cassava fields in the environs of the National Crops Resources Research Institute (NaCRRI) in Namulonge, Uganda. The Nam 130 and Nase 14 are cassava cultivars, and we included a local variety, Njule Red, later in the study–we refer to all these as “varieties” throughout. The two source varieties are currently widely adopted in farmers’ fields. Because populations of *B*. *tabaci* build up and drop as cassava matures [37], different cassava age classes were used. Although we could identify varieties by their known physical characteristics, the age was confirmed by asking the farmers for the planting dates. Both Nam 130 and Nase 14 are improved varieties, with high and moderate resistance to CMD, respectively [25]. We used five replicate fields of each variety by age treatment for a total of 30 fields, which were distributed haphazardly across a 5 km × 5 km area.

Sentinel plants (potted cassava plants) of two varieties (Nam 130 and Nase 14) and a landrace (Njule Red, which is susceptible to CMD) were propagated in a screen house from virus-free stem cuttings until they were three weeks old. These three genotypes were chosen because they are commonly grown in farmers’ fields in Uganda, and have varying levels of resistance to CMD and CBSD. Plants were grown in 20-cm diameter pots (5 L). All pots (for each of the three varieties) were of a similar color to avoid potential confounding effects if *B*. *tabaci* had preferential responses to color [38–40]. Pots were filled with loam soil and watered every two days. The screen house prevented oviposition by *B*. *tabaci* prior to the experiment commencing.

### Abundance of *B*. *tabaci* on source and sentinel plants

In the source fields, counts of adult *B*. *tabaci* were taken before sentinel plants were exposed. The counts were taken on the top five fully-expanded leaves according to a published method [41]. We sampled from 30 plants in each of the 30 fields. The cassava plants were free of any disease symptoms.

In each source field, two potted sentinel plants of each variety were placed for one week to allow colonization and oviposition. The sentinel plants were placed 2 m from each other. The sentinel plants were then removed from the field and incubated separately in rectangular screened cages (1 m × 1 m × 1.5 m) for three weeks to allow development of eggs to third or fourth instar nymphs. All leaves were examined using a binocular microscope (Wild Heerbrugg, Switzerland) at 80× magnification and all nymphs counted.

### Species identity of *B*. *tabaci* species complex

A sample of whitefly individual specimens (four sites randomly selected) was genotyped using a PCR-based protocol. The DNA was extracted from a total number of 40 individuals (10 per site) using the Qiagen Blood and Tissue DNA extraction kit (Cat. # 69056) following the recommended protocol and its quality ascertained using Qubit 2 Fluorometer (Life Technologies Corporation). Samples with Qubit fluorometer gDNA concentration >0.05 ng/μL of gDNA were used in PCR. The PCR amplification of whitefly partial mitochondrial DNA cytochrome c oxidase subunit I (mtDNA COI) gene used the primers as reported in Elfekih et al. [42]. The PCR was performed according to Elfekih et al. [42] but with modifications as follows: incubation at 95°C for 5 min; 37 cycles of incubation at 95°C for 60 s, at 54°C for 30 s and at 72°C for 45 s; with a final incubation at 72°C for 5 min, and post PCR incubation at 10°C. The PCR products were electrophoresed in 1.25% agarose gel and PCR amplicons sent for sequencing. The DNA trace files for individual samples were analyzed and contigs assembled using the PreGap4 and Gap4 programs within the Staden package for DNA sequence assembly, editing, and analysis [43]. Assembled partial mtDNA COI sequences were searched using BLAST [44] against non-redundant reference sequences in the NCBI database and whiteflybase [45] to confirm their species identity.

### Data analysis

To address our first research question “Does variety or plant age influence *B*. *tabaci* density?”, we used adult count data per plant collected prior to the experiment starting which was log(x + 1)-transformed to stabilize the variance. We used generalized linear mixed effects models to test the relationship between adult counts prior to the beginning of the experiment and two factors: the cassava variety in the source field (two levels: Nam 130 and Nase 14) and cassava age in the source field (three levels: young, medium, and old). To test for differences in the density of adult *B*. *tabaci* per plant, we used plant-level data from 30 fields (five replicate fields of each treatment combination) and included the factor ‘field’ as a random effect. Thus, our model was:

$${\mathrm{Density}}_{\mathrm{Source}}{=\mathrm{Variety}}_{\mathrm{Source}}{+\mathrm{Age}}_{\mathrm{Source}}+\left({\mathrm{Variety}}_{\mathrm{Source}}{\;:\;\mathrm{Age}}_{\mathrm{Source}}\right),\;\mathrm{random}=∼1|\mathrm{field}$$

We tested different models with all combinations of the fixed factors and interaction to find the best fitting model. We compared models by performing the likelihood ratio test using ANOVA, and selected the best model based on Akaike information criterion (AIC) values. Variance was estimated by the maximum likelihood (ML) method with a negative binomial distribution. We checked the final model for normality, heterogeneity, and independence by graphing the residuals. We used the ‘lme’ function in the ‘nlme’ package [45] in R.

To examine the relationships related to our second research question “Does *B*. *tabaci* exhibit colonization preferences based on their source plant?”, we used generalized linear mixed effects models to test the significance of the main effects (and selected interactions). The response variable was the density of *B*. *tabaci* nymphs (third to fourth instars) on the two sentinel plants in each field. The explanatory variables were whitefly adult density at source at the start of the experiment (range 0–546), cassava variety at the source (two levels: Nam 130 and Nase14), cassava age (three levels: young, medium, and old), and the cassava variety used as the sentinel plant. The factor ‘field’ was included as a random effect, and variance was estimated by the ML method. We included the interaction term (Variety_source_: Variety_sentinel_) to test our third hypothesis “*B*. *tabaci* prefer to colonize a familiar variety”. Thus, our final model was:

$${\mathrm{Density}}_{\mathrm{Sentinel}}{=\mathrm{Density}}_{\mathrm{Source}}{+\mathrm{Age}}_{\mathrm{Source}}{+\mathrm{Variety}}_{\mathrm{Sentinel}}+\left({\mathrm{Variety}}_{\mathrm{Source}}{\;:\;\mathrm{Variety}}_{\mathrm{Sentinel}}\right),\;\mathrm{random}=∼1|\mathrm{field}$$

We tested eight different models including different combinations of the explanatory variables using the ‘lme’ function [47]. We then performed multiple comparisons using the ‘multicomp’ function [48] and selected for the degree of fit of our models based on their AIC values. We checked the final model for normality, heterogeneity, and independence by graphing the residuals. We found that one source field had very high numbers of adults prior to the experiment starting so we repeated the analysis above without this source field and presented results with the complete data set (Table 1) and a reduced data set (S1 Table). Significant differences between levels of factors were tested using the Tukey post-hoc test in the ‘multicomp’ package [48] in R.

**Table 1:** The final, best fitting, linear mixed effects models to test which factors influence colonization preferences of *Bemisia tabaci*. The table for the model is based on the full dataset.

Factor	Df	*F*-value	P-value
**Intercept**	1, 145	314.04	**<0.0001**
**Density of adults at source**	1, 25	4.40	**0.0463**
**Age of source field**	2,25	2.01	0.1547
**Source cultivar**	1,25	0.83	0.3706
**Sentinel cultivar**	2,145	2.33	0.1005
**Source cultivar : Sentinel cultivar interaction**	2, 145	6.10	**0.0029**

R-sq. (fixed effects) = 0.23

## Results

### Analysis of *B*. *tabaci* species complex

Four individual extractions had <0.05 ng/μL of gDNA. Of the remaining 36 individuals from the sampling sites, PCR amplification produced the anticipated fragment size of ca. 780 bp [42]. We randomly selected 12 individuals representing three adult whiteflies per site for sequencing. The BLAST search results against NCBI and whiteflybase reference sequences confirmed that the *B*. *tabaci* cryptic species complex prevalent in the experimental area was the *B*. *tabaci* SSA1 species. The sequences of the 12 individuals are available in GenBank (accession numbers: MH410701–MH4107012). Three mtDNA COI haplotypes were detected from these 12 individual sequences of which two represented novel haplotypes of SSA1 (GenBank accession numbers: MH410708 and MH4107012).

### Density of *B*. *tabaci* as a function of plant age or variety

**Fig 1:**
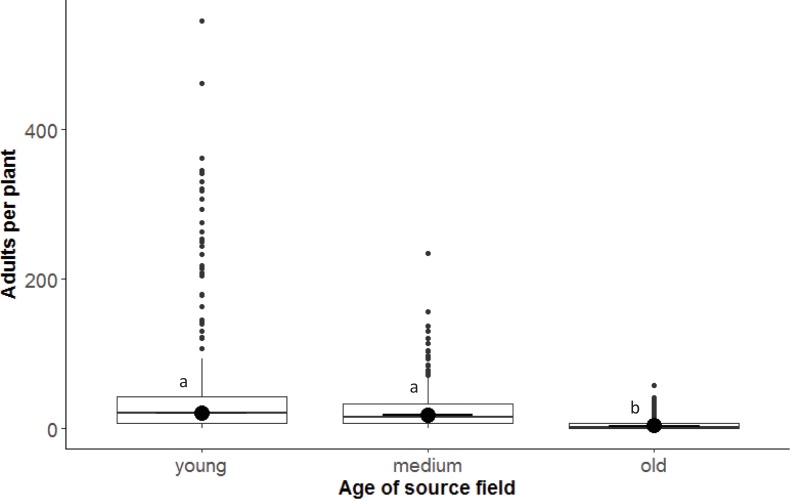
Box-and-whisker plots of the density of adult *Bemisia tabaci* on cassava plants in fields sown with two cassava varieties (Nam 130 and Nase 14) at different ages: Young (2–3 m.a.p.), medium (4–6 m.a.p.), old (7–9 m.a.p.). Data were collected prior to the exposure of sentinel plants.

Prior to beginning the experiment, the density of adult *B*. *tabaci* differed as a factor of source age (F = 8.32; df = 2, 27; P < 0.001) (Fig 1) but not source variety (P = 0.465). Adult density on old cassava plants was significantly lower than on both young (P < 0.001) and medium (P = 0.0018) age plants. The number of adult *B*. *tabaci* per plant, in general, ranged within 0–546.

#### Density of *B*. *tabaci* on source plants

**Fig 2:**
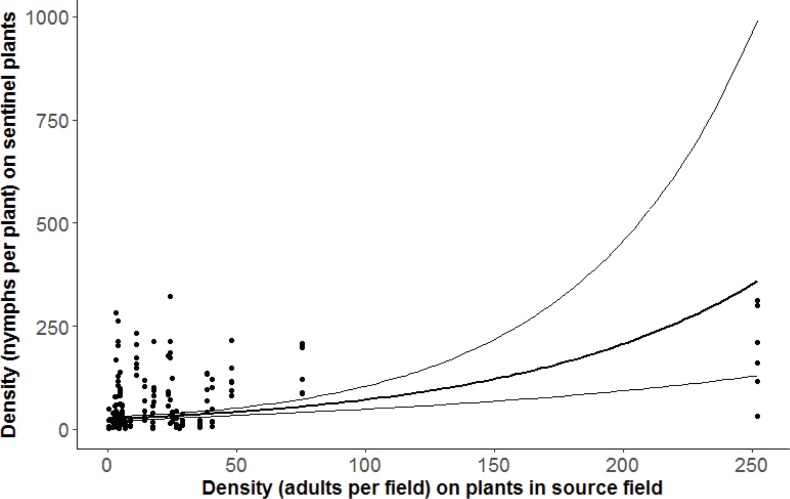
The density of *B*. *tabaci* nymphs in the source field plotted against density of *B*. *tabaci* nymphs on the sentinel cassava plants. Fit of general linear mixed effects model shown (log-transformed response).

We found a significant positive relationship (F = 4.40, df = 1, 25, P = 0.0463) between the density of *B*. *tabaci* on sentinel plants and the density of *B*. *tabaci* on source plants (Fig 2, Table 1), which supports our first hypothesis. However, this pattern was heavily dependent on one field in which we found very high densities (more than five times) of *B*. *tabaci* on source plants (Fig 2). When this field was removed from the analysis, there was no longer a significant relationship (F = 2.92, df = 1, 24; P = 0.1006) between density of nymphs on sentinel plants and density of adults in the source field (S1 Table). Thus, the first hypothesis was not supported in the reduced dataset.

#### Age of source field

**Fig 3:**
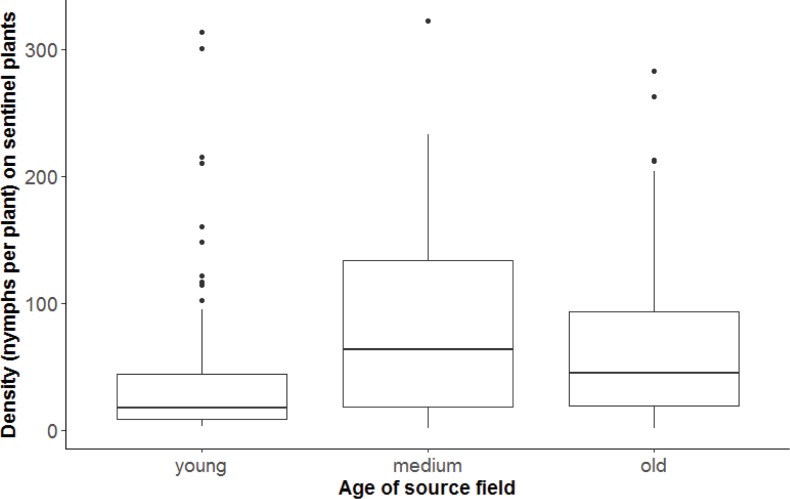
Box-and-whisker plots of the density of *B*. *tabaci* nymphs on sentinel plants and the age of the source field. No significant differences between the ages.

Density of *B*. *tabaci* on sentinel plants was not significantly related to source age (F = 2.01, df = 2, 25; P = 0.1547) (Fig 3, Table 1). If we again used the reduced dataset that removed the source field with very high densities (age category = young), the age of the source had no significant effect on the colonization of sentinel plants (F = 2.49, df = 2, 24; P = 0.1041) (S1 Table).

#### Variety of source field

**Fig 4:**
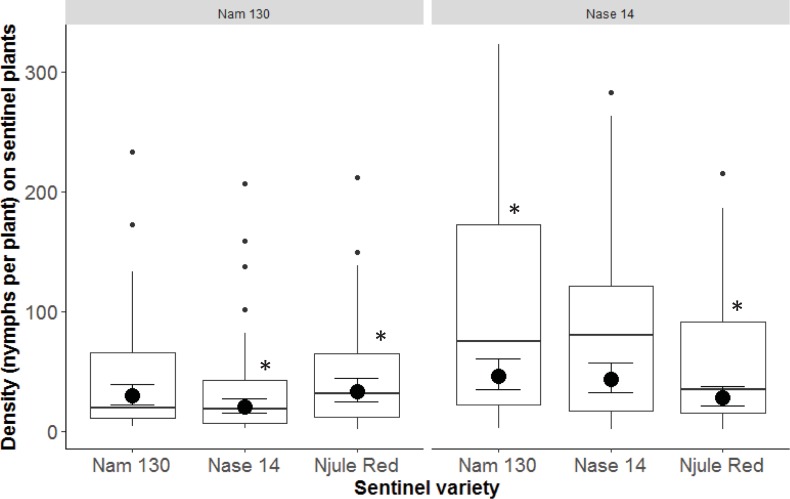
Plot showing as large points the modeled mean density of *B*. *tabaci* nymphs on sentinel plants of each variety as a function of the source variety (left, Nam130; right, Nase14). Confidence intervals show the standard error of the model. Raw data are also plotted (small points). * denotes significant difference between two sentinel varieties within a source variety.

The *B*. *tabaci* adults did not preferentially colonize a sentinel plant of the same variety as the source field (Fig 4, Table 1). We saw no evidence of a preference for adults to move from a source variety to the same sentinel variety and oviposit. However, there was a positive interactive effect between source variety and sentinel variety (F = 6.10, df = 2, 145, P = 0.0029, Table 1). The nymph density on Nase 14 sentinels was (marginally) significantly lower than on Njule Red sentinels when Nam 130 was the source (P = 0.08); and there was also a significantly higher nymph density on Nam 130 sentinels compared to Njule Red when Nase 14 was the source (P = 0.030) (Tukey posthoc test).

## Discussion

This manipulative field experiment showed that fields of different ages and cassava varieties supported different densities of *B*. *tabaci* SSA1 cryptic species and therefore will differ in their ability to act as sources of colonizers to newly planted cassava fields. We found a positive relationship between the density of nymphs on sentinel plants and the density of adult *B*. *tabaci* in the source field (Fig 2). This has important implications for management; planting near sources with high whitefly density puts new crops at greater risk and should be avoided.

If the sources also have a high incidence of CMD and CBSD, the risk would even be greater, because the migrant whiteflies are likely to be viruliferous and hence spread the diseases. There is evidence that the density of mixed crops affects the distribution of disease incidence and that differences in disease incidences may be mainly due to changes in size of the whitefly population [49]. Mixed crops may involve two or more crops simultaneously intercropped with cassava in smallholder farms, but intercropping may broadly include mixtures of crop cultivars as well [50]. In addition, multiple cropping systems are known to affect insect dynamics at various stages including colonization of crops, development of populations, and dispersal and abundance of natural enemies [51]. For example, cassava intercropped with maize in Ivory Coast reduced *B*. *tabaci* population abundances [52] and cassava intercropped with beans in Colombia had a similar effect [53]. In Tanzania, mung bean intercropped with two cassava cultivars not only reduced *B*. *tabaci* populations significantly but also reduced the incidence and severity of CMD [54].

Little research has investigated how different cassava cultivars might influence the activities of natural enemies of *B*. *tabaci*. Legg [55] found significantly higher numbers of *B*. *tabaci* and parasitoids on a CMD-resistant cultivar compared to a susceptible cultivar. It is also known that cassava cultivars of different morphologies can influence predators such as *Typhlodromalus aripo* that also preys on the cassava green mite *Mononychellus tanajoa* [56]. Souissi & Le Rü [57] studied the relationship between the cassava plant and the parasitoid *Apoanagyrus* (*Epidinocarsis*) *lopezi* De Santis (Hymenoptera: Encyrtidae) used in the biological control of *Phenacoccus manihoti* in Africa and found that cassava with a high level of antibiosis resistance had a deleterious effect on *A*. *lopezi* survival and development. It would be expected that because young- and medium-aged fields hosted high *B*. *tabaci* densities, they would equally host a higher number of natural enemies compared with old fields; however, there is no research evidence available. Thus, a comprehensive understanding of cultivar and age impacts on *B*. *tabaci* natural enemy dynamics is needed.

We predicted that sentinel plants would have higher colonization rates in medium age fields; however, colonization of sentinel plants did not significantly differ with age (P = 0.1547) (Fig 3). A possible reason for this may be that adults in young source fields were unlikely to move because resource quality was still sufficiently good to support their development and fitness, whereas as host plants increase in age, their quality as a resource declines [23]. In theory, adults are more likely to move and colonize new plants once resources in their current field diminish past a certain threshold; however, the exact nature of such a threshold has not been determined and is likely to vary by variety. There also may be density-dependent movement in which high densities in the source crop may promote migration away from it.

Contrary to our expectations, *B*. *tabaci* SSA1 adults did not preferentially colonize sentinel plants of the same variety as the source field; there was no significant difference between the densities on sentinel varieties that matched the source variety, compared with other sentinel varieties (Fig 4). We thus conclude that there was no evidence of *B*. *tabaci* preference to move to the same cultivar from the source, meaning that risk was similar across varieties when comparing within source group. Although variety responses to whitefly abundance have been recognized [58], no relationships between some plant variety traits such as leaf area and width and the numbers of *B*. *tabaci* have been found [59]. A variety with low numbers of *B*. *tabaci* was, contrary to expectations, found to have a higher sooty mold severity score, a factor attributed to the broader leaves [58]. There is also evidence to suggest that plant allellochemicals play a role in determining suitability for growth and development of *B*. *tabaci* populations [59,60]. Further studies are needed to investigate the mechanisms underlying these observations.

These combined results indicate that source variety is more important for colonization dynamics than the sentinel variety and should be the focus of future management actions. Thus, planting a “whitefly sink” variety (one that would preferentially be colonized by *B*. *tabaci*) is unlikely to reduce *B*. *tabaci* SSA1 populations, without critical management to reduce densities on the source variety at the same time. Whitefly-susceptible varieties (those that support both high adult and nymph populations) should be avoided if *B*. *tabaci* SSA1 populations are to be controlled.

A limitation of our study was the use of only three of the several hundreds of cassava varieties among smallholder farms in the Eastern Africa region [61]. Both Nam 130 and Nase 14 are improved varieties whereas Njule Red is a local landrace. Nam 130 and Nase 14 have high and moderate resistance to CMD, respectively [25], and Njule Red is susceptible; however, all three varieties were very susceptible to *B*. *tabaci* colonization. Breeding cassava cultivars has been the main approach to managing cassava viral diseases, but these improved varieties are very susceptible to *B*. *tabaci*. Hence, future breeding efforts need to combine not only resistance breeding for both the disease and the vector but also the development of integrated approaches to control *B*. *tabaci* also making use of natural enemies [23]. Future studies should include additional varieties relevant to the region of interest, which may further demonstrate the importance and impact of individual varieties in determining *B*. *tabaci* preferences. The role of other host plants was a factor that we did not investigate–*B*. *tabaci* SSA1 adults may have moved from sources other than the identified source field. For example, *Euphorbia* weeds and any other crop and non-crop hosts available in the surrounding landscape may also act as a source host. Because landscape factors may vary in composition, configuration, and suitability, their influence as sources of *B*. *tabaci* and on colonization dynamics on cassava need to be evaluated. Our study also did not take into account any other factors that could have resulted in *B*. *tabaci* mortality between egg laying and development of the nymph stage, which may have also influenced the results.

### Conclusions

A number of factors are known to influence the behavior of invertebrates, their potential for spread, and the level of damage they inflict on plants; understanding these factors can lead to improved management options [62–65]. For smallholder cassava farmers, there is great value in understanding what management strategies can reduce plant damage due to the cryptic species complex associated with *B*. *tabaci*. Our results demonstrated, firstly, that age of the cassava source plant can determine the risk for nearby plants of being colonized by *B*. *tabaci* SSA1. Secondly, there was a positive correlation between the density of nymphs on sentinel plants and the density of adults in the source field; and, thirdly, there was a significant interactive effect between the source and sentinel varieties that may indicate variability in colonization. Future work should investigate the planting timing of new cassava and how to prioritize varieties that both have traits that farmers desire and also host lower densities of *B*. *tabaci* SSA1. Studies involving other members of the *B*. *tabaci* cryptic species complex such as SSA2, MED, and Indian Ocean [14–16], which to a certain extent can establish populations on cassava, should be undertaken to understand how such species could be managed on cassava.

## Supporting information

S1 Table The final, best fitting, linear mixed effects models to test which factors influence the colonization preferences of *Bemisia tabaci*. The table is based on the reduced dataset, removing a field that had an unusually high density of *B*. *tabaci* at the start of the experiment.
(DOCX)

S1 File Model descriptions in R. (DOCX)
