# Supplementary material for: African cassava whitefly, Bemisia tabaci, cassava colonization preferences and control implications
Source: PLoS One. 2018 Oct 9;13(10):e0204862. doi: 10.1371/journal.pone.0204862 (PMC6177144; doi:10.1371/journal.pone.0204862)
Supplement: S1 Table — The table is based on the reduced dataset, removing a field that had an unusually high density of B. tabaci at the start of the experiment. (DOCX) [file pone.0204862.s001.docx]

| Factor | Df | *F*-value | P-value |
| --- | --- | --- | --- |
| Intercept | 1, 140 | 301.79 | **<0.0001** |
| Density of adults at source | 1, 24 | 2.92 | 0.1006 |
| Age of source field | 2, 24 | 2.49 | 0.1041 |
| Source cultivar | 1, 24 | 0.63 | 0.4357 |
| Sentinel cultivar | 2,140 | 1.70 | 0.1860 |
| Source cultivar: Sentinel cultivar interaction | 2, 140 | 5.54 | **0.0048** |

R-sq. (fixed effects) = 0.22
