# Supplementary material for: African cassava whitefly, Bemisia tabaci, cassava colonization preferences and control implications
Source: PLoS One. 2018 Oct 9;13(10):e0204862. doi: 10.1371/journal.pone.0204862 (PMC6177144; doi:10.1371/journal.pone.0204862)
Supplement: S1 File — (DOCX) [file pone.0204862.s002.docx]

#CLEAN R SCRIPT USED FOR STATS AND GRAPHS JUNE 2018

#load packages

library(plyr)

library(ggplot2)

library(multcomp)

library(effects)

#Import data -

prior<-read.csv("source prior.csv", header=TRUE)

#Check the format for each column is correct (i.e. should it be a factor or a number?)

prior$field<-as.factor(prior$field)

str(prior)

#set the levels of the "age" factor

prior$age <- factor(prior$age, levels = c("young", "medium", "old"))

sourcedata <-prior

#Read in data set of the potted plant data

potted<-read.csv("colonization potted plants.csv", header=TRUE)

#Check the format for each column is correct (i.e. should it be a factor or a number?)

potted$field<-as.factor(potted$field)

str(potted)

#set the levels of the "age" factor

potted$age <- factor(potted$age, levels = c("young", "medium", "old"))

#STATISTICS AND RELEVANT GRAPHS

#H1a - adult density on SOURCE PLANTS

#glmer approach

library(DHARMa)

library(lme4)

library(effects)

library(blmeco)

library(MuMIn)

m3<-glmer(wfadult ~ source*age+(1|field), data=prior,family=poisson)

summary(m3)

dispersion_glmer(m3)

m4<-glmer.nb(wfadult ~ source*age+(1|field), data=prior)

dispersion_glmer(m4)

AIC(m3,m4)

summary(m4)

m5<-glmer.nb(wfadult ~ source+age+(1|field), data=prior)

AICc(m4,m5)

summary(m5)

m6<-glmer.nb(wfadult ~ age+(1|field), data=prior)

AICc(m5,m6)

summary(m6)

anova(m6)

#THE BEST FIT MODEL IS m6, where age is the only significant factor.

#Other models do not fit so well and other factors were not found to be significant.

simulationOutput <- simulateResiduals(fittedModel = m6, n=1000)

plotSimulatedResiduals(simulationOutput = simulationOutput)

plotSimulatedResiduals(simulationOutput = simulationOutput, asFactor =T)

testUniformity(simulationOutput)

testZeroInflation(simulationOutput)

library(multcomp)

#TESTING FOR SIGNIFICANT DIFFERENCES (POSTHOC TUKEY TEST)

summary(glht(m6, linfct=mcp(age="Tukey")))

fig1<-ggplot(efdf, aes(x=age, y=fit))+

geom_boxplot(data = prior, aes(x=age, y = wfadult)) +

geom_point(size = 5) +geom_errorbar(limits, width=0.4) +

xlab("Age of source field") + ylab("Adults per plant")+

theme(axis.text=element_text(size=14),

axis.title=element_text(size=14,face="bold"))+

theme (panel.background =element_blank(), axis.line = element_line(color = "black"))

fig1

#H1b - nymph density on SENTINEL PLANTS

#use datasets with mean per field for source, and mean per plant for sentinel.

sourcedata <- ddply(prior, .(age, source, field), summarise,

mean = mean(wfadult, na.rm=TRUE))

head(sourcedata)

sentineldata <- ddply(potted, .(age, source, field, sentinel, plant), summarise,

mean = mean(nymph, na.rm=TRUE))

head(sentineldata)

names(sentineldata)[names(sentineldata) == 'mean'] <- 'mean.sentinel'

names(sourcedata)[names(sourcedata) == 'mean'] <- 'mean.source'

mergedata<- merge(sourcedata,sentineldata,by=c("age","source","field"),all.x=TRUE,all.y=TRUE)

head(mergedata)

require(nlme)

#Testing appropriate random structure on the full model (NB compare with REML)

m3<-lme(mean.sentinel ~ mean.source+age + source * sentinel

,random=~1|field,method="REML", data=mergedata,na.action=na.omit)

m4<-lme(mean.sentinel ~ mean.source+age + source * sentinel

,random=~1|field/sentinel,method="REML", data=mergedata,na.action=na.omit)

AIC(m3,m4)

anova(m3,m4)#m3 is fine, no real difference so going with simpler structure

summary(m3)

E<-resid(m3, type="normalized")

Fit<-fitted(m3)

qqnorm(E)

plot(E)

plot(x=Fit,y=E,xlab="fitted values",ylab="residuals",main="residuals vs fitted values")

#not a great fit - pattern at low fitted values, try transforming by log(mean.sentinel+1), compare with ML

#not using poisson or neg bin as not count data (using densities)

m3<-lme(mean.sentinel ~ mean.source+age + source * sentinel

,random=~1|field,method="ML", data=mergedata,na.action=na.omit)

m3a<-lme(log(mean.sentinel+1) ~ mean.source+age + source * sentinel

,random=~1|field,method="ML", data=mergedata,na.action=na.omit)

AIC(m3,m3a)

summary(m3a)

E<-resid(m3a, type="normalized")

Fit<-fitted(m3a)

qqnorm(E)

plot(E)

plot(x=Fit,y=E,xlab="fitted values",ylab="residuals",main="residuals vs fitted values")

#not bad, could be better (heteroscedacity), try weights for better fit

#NB use ML not REML for model to model comparison (fixed structure)

m5<-lme(log(mean.sentinel+1) ~ mean.source+age + source * sentinel

,random=~1|field,method="ML",

data=mergedata,na.action=na.omit,

weights=varIdent(form=~1|age))

m6<-lme(log(mean.sentinel+1) ~ mean.source+age + source * sentinel

,random=~1|field,method="ML",

data=mergedata,na.action=na.omit,

weights=varIdent(form=~1|source))

m7<-lme(log(mean.sentinel+1)~ mean.source+age + source * sentinel

,random=~1|field,method="ML",

data=mergedata,na.action=na.omit,

weights=varIdent(form=~1|sentinel))

m8<-lme(log(mean.sentinel+1) ~ mean.source+age + source * sentinel

,random=~1|field,method="ML",

data=mergedata,na.action=na.omit,

weights=varIdent(form=~1|sentinel*source))

m9<-lme(log(mean.sentinel+1) ~ mean.source+age + source * sentinel

,random=~1|field,method="ML",

data=mergedata,na.action=na.omit,

weights=varPower(form=~mean.source))

m10<-lme(log(mean.sentinel+1) ~ mean.source+age+source * sentinel

,random=~1|field,method="ML",

data=mergedata,na.action=na.omit,

weights=varIdent(form=~1|age*source))

AIC(m3a,m5,m6,m7,m8,m9,m10)

anova(m3a,m7)

#after all the testing this model is best:

m7<-lme(log(mean.sentinel+1)~ mean.source+age + source * sentinel

,random=~1|field,method="REML",

data=mergedata,na.action=na.omit,

weights=varIdent(form=~1|sentinel))

E<-resid(m7, type="normalized")

Fit<-fitted(m7)

qqnorm(E)

plot(E)

plot(x=Fit,y=E,xlab="fitted values",ylab="residuals",main="residuals vs fitted values")

summary(m7)

anova(m7)

#compute r2: marginal = r2 for fixed effects, conditional = includes random effects

r.squaredGLMM(m7)

# Generating Table 1 with reduced data set-outlier field removed

mergedata1<-mergedata[mergedata$mean.source<200,]

m7b<-lme(log(mean.sentinel+1)~ mean.source+age + source * sentinel

,random=~1|field,method="REML",

data=mergedata1,na.action=na.omit,

weights=varIdent(form=~1|sentinel))

E<-resid(m7b, type="normalized")

Fit<-fitted(m7b)

qqnorm(E)

plot(E)

plot(x=Fit,y=E,xlab="fitted values",ylab="residuals",main="residuals vs fitted values")

summary(m7b)

anova(m7b)

r.squaredGLMM(m7b)

#this is basically the same model but with new interaction factor to explore interaction effects

mergedata$new.factor=paste(mergedata$source,mergedata$sentinel)

mergedata$new.factor=as.factor(mergedata$new.factor)

mergedata <- within(mergedata, new.factor <- relevel(new.factor, ref = "Nase 14 Njule Red"))

m7a<-lme(log(mean.sentinel+1) ~ mean.source+age+new.factor

,random=~1|field,method="REML",

data=mergedata,na.action=na.omit,

weights=varIdent(form=~1|sentinel))

E<-resid(m7a, type="normalized")

Fit<-fitted(m7a)

qqnorm(E)

plot(E)

plot(x=Fit,y=E,xlab="fitted values",ylab="residuals",main="residuals vs fitted values")

summary(m7a)

anova(m7)

levels(mergedata$new.factor)

#"Nam 130 Nam 130" "Nam 130 Nase 14" "Nam 130 Njule Red"

#[4] "Nase 14 Nam 130" "Nase 14 Nase 14" "Nase 14 Njule Red"

#TESTING FOR SIGNIFICANT DIFFERENCES (POSTHOC TUKEY TEST)

summary(glht(m7, linfct=mcp(age="Tukey")))

summary(glht(m7a, linfct=mcp(new.factor="Tukey")))

#test for multi-colinarity in data threshold should be below 2 for no multi colinearity

vif.mer <- function (fit) {

## adapted from rms::vif

v <- vcov(fit)

nam <- names(fixef(fit))

## exclude intercepts

ns <- sum(1 * (nam == "Intercept" | nam == "(Intercept)"))

if (ns > 0) {

v <- v[-(1:ns), -(1:ns), drop = FALSE]

nam <- nam[-(1:ns)]

}

d <- diag(v)^0.5

v <- diag(solve(v/(d %o% d)))

names(v) <- nam

v

}

vif.mer(m7)

#GRAPHS OF RESULTS

summary (m7a)

plot(allEffects(m3a))

ef<-effect("mean.source",m3a,xlevels=100)

ef

efdf<- as.data.frame(ef)

efdf

efdf$max <- efdf$fit +efdf$se

efdf$min <- efdf$fit -efdf$se

#graph with ggplot2, showing the model

fig2<-ggplot(mergedata, aes(x=mean.source, y=mean.sentinel)) +

geom_point()+ xlab("Mean density adults on plants in source field") + ylab("Mean density nymphs on sentinel plants")+

theme (panel.background = element_blank(), axis.line =element_line(color ="black"), axis.text=element_text(size=14),

axis.title=element_text(size=14))+

geom_line(data = efdf, aes(x=mean.source, y = exp(fit)-1), size = 1)+

geom_line(data = efdf, aes(x=mean.source, y = exp(max)-1), size = 0.5) +

geom_line(data = efdf, aes(x=mean.source, y = exp(min)-1), size = 0.5)#+ geom_errorbar(limits, width=0.4))

fig2

fig3<-ggplot(sentineldata, aes(x=age, y=mean.sentinel)) +

# geom_boxplot(outlier.colour="red", outlier.shape=8,outlier.size=4) +

geom_boxplot() +

xlab("Age of source field") + ylab("Mean density nymphs sentinel plants")+

theme(axis.text=element_text(size=14),

axis.title=element_text(size=14,face="bold"))+

theme (panel.background =element_blank(), axis.line = element_line(color = "black")) #+ylim(0,300)

fig3

ef<-effect("source:sentinel",m3a,xlevels=100)

efdf<- as.data.frame(ef)

efdf

efdf$max <- efdf$fit +efdf$se

efdf$min <- efdf$fit -efdf$se

limits <- aes(ymin=exp(fit-se)-1, ymax=exp(fit+se)-1)

fig4<-ggplot(efdf, aes(x=sentinel, y=exp(fit)-1))+ #+ylim(0,300)+

geom_boxplot(data = sentineldata, aes(x=sentinel, y = mean.sentinel)) +

geom_point(size = 5) +geom_errorbar(limits, width=0.4) +

facet_grid(~source)+

xlab("Sentinel variety") + ylab("Mean density nymphs sentinel plants")+

theme(axis.text=element_text(size=14),

axis.title=element_text(size=14,face="bold"))+

theme(panel.background =element_blank(), axis.line = element_line(color = "black"))

fig4
